# Honoring our teachings: children’s storybooks as indigenous public health practice

**DOI:** 10.3389/fpubh.2024.1354761

**Published:** 2024-02-23

**Authors:** Tara L. Maudrie, Fiona Grubin, Maisie Conrad, Jocelyn Velasquez Baez, Jessica Saniguq Ullrich, Joshuaa Allison-Burbank, Lisa Martin, Crystal Austin, Joelle Joyner, Marcella Ronyak, Kristin Masten, Allison Ingalls, Emily E. Haroz, Victoria M. O’Keefe

**Affiliations:** ^1^Johns Hopkins Center for Indigenous Health, Baltimore, MD, United States; ^2^Department of Molecular Biology and Biochemistry, Wesleyan University, Middletown, CT, United States; ^3^Institute for Research and Education to Advance Community Health (IREACH), Washington State University, Spokane, WA, United States; ^4^Department of Public Health, Wayne State University, Detroit, MI, United States; ^5^Indian Health Service, Rockville, MD, United States

**Keywords:** culturally grounded, American Indian/Alaska native, storytelling, COVID-19, indigenous research

## Abstract

**Introduction:**

American Indian and Alaska Native (AIAN) communities continue to flourish and innovate in the face of the COVID-19 pandemic. Storytelling is an important tradition for AIAN communities that can function as an intervention modality. To support the needs of AIAN children and caregivers, we (a collaborative workgroup of Indigenous health researchers) developed a culturally grounded storybook that provides pandemic-related public health guidance and mental health coping strategies woven with Inter-Tribal values and teachings.

**Methods:**

A collaborative workgroup, representing diverse tribal affiliations, met via four virtual meetings in early 2021 to discuss evolving COVID-19 pandemic public health guidance, community experiences and responses to emerging challenges, and how to ground the story in shared AIAN cultural strengths. We developed and distributed a brief survey for caregivers to evaluate the resulting book.

**Results:**

The workgroup iteratively reviewed versions of the storyline until reaching a consensus on the final text. An AI artist from the workgroup created illustrations to accompany the text. The resulting book, titled Our Smallest Warriors, Our Strongest Medicine: Honoring Our Teachings during COVID-19 contains 46 pages of text and full-color illustrations. An online toolkit including coloring pages, traditional language activities, and caregiver resources accompanies the book. We printed and distributed 50,024 physical copies of the book and a free online version remains available. An online survey completed by *N* = 34 caregivers who read the book with their child(ren) showed strong satisfaction with the book and interest in future books.

**Discussion:**

The development of this storybook provides insights for creative dissemination of future public health initiatives, especially those geared toward AIAN communities. The positive reception and widespread interest in the storybook illustrate how braiding AIAN cultural teachings with public health guidance can be an effective way to disseminate health information. This storybook highlights the importance of storytelling as an immersive learning experience through which caregivers and children connect to family, community, culture, and public health guidance. Culturally grounded public health interventions can be effective and powerful in uplifting AIAN cultural values and promoting health and well-being for present and future generations.

## Introduction

American Indian and Alaska Native (AIAN) peoples are the original stewards of Turtle Island, including the so-called United States (U.S.), and share a long history of resistance and self-determination in the face of ongoing colonization and structural racism. There are currently 574 federally recognized AIAN tribes and additional state-recognized tribes, all of which are ethnically, culturally, and linguistically diverse (1). Centuries of ongoing structural oppression have led to inadequate education and health care, poverty, and disproportionate burden of disease for many AIAN communities (2, 3). Limited access to health care is also a challenge for many Tribal communities located in rural areas, and urban AIANs who have limited health care funding (4–6). Some AIAN peoples may also be hesitant to seek health care services due to historical and contemporary mistreatment by government institutions and health care systems (7). Together these factors led to the devastating and disparate impact of the COVID-19 pandemic in many AIAN communities. Morbidity and mortality rates due to COVID-19 among AIAN peoples were some of the highest in the nation at various points during the pandemic, despite AIANs making up only 2.9% of the U.S. population (3, 8). In the early stages of the pandemic, physical distancing and lockdowns disrupted cultural practices and ceremonies that are vital to the spiritual and mental health of AIANs (9). Additionally, the loss of Elders and other loved ones had devastating impacts on AIAN mental health (10). Given the increased burden and associated impacts on family, community, and ability to practice cultural traditions, it is not surprising that increased rates of depression, anxiety, and stress challenged many AIAN communities during the pandemic (11).

The pandemic also presented significant disruptions to the lives of many AIAN children and their caregivers. Complex and rapidly evolving COVID-19 mitigation measures caused changes to education, daily routines, and social connection for children (12). Closures and shifts to virtual and hybrid school and community programming changed access to school-based services, peer support, and social interaction with others beyond their household (13). Children are particularly susceptible to the mental health impacts of events like the pandemic due to developmental stages and limited cognitive ability to understand complex situations, to independently develop and use coping strategies, and to adequately communicate their feelings (12). In addition to new and exacerbated stressors from the pandemic, caregivers navigated communicating complicated information about the rapidly evolving pandemic with their children (14).

Despite facing escalating challenges during the COVID-19 pandemic, AIAN communities continue to flourish and innovate to meet community needs. Strong kinship networks and community-driven values foster mutual support and care when navigating challenges and this was especially present during the pandemic (15). Prompted by increased media attention on AIAN communities during the pandemic, many Native-led organizations continue to advocate for structural and policy reforms and to raise public awareness of ongoing health inequities AIANs face (16). Tribal Nations demonstrated sovereignty and tailored and implemented local COVID-19 prevention responses to meet the urgent needs of their communities (17). For example, some communities established mutual aid networks to provide services such as patient transport, household food delivery, and distribution of personal protective equipment (17). Due to community-led and culturally grounded vaccination efforts, AIANs have consistently had the highest COVID-19 vaccination rates of any racial or ethnic groups in the United States (18, 19).

Intergenerational knowledge, such as the passing on of Tribal languages, traditional ecological knowledge, oral storytelling, and cultural teachings, plays an important role in cultural and holistic well-being for many AIAN communities (20, 21). Storytelling in many AIAN communities is a traditional and dynamic way of sharing knowledge, entertainment, and cultural teachings that can be harnessed as part of interventions to promote well-being (22). Multiple health promotion programs have incorporated culturally relevant storybooks to engage AIAN children through storytelling and promote positive health outcomes (23). Indigenous communities across the Earth also engage in storytelling to support well-being. For example, researchers in Canada created storybooks to help facilitate healing and reduce mental health distress stemming from intergenerational colonial trauma (24). In Australia, art and storytelling represent important communication and knowledge transmission traditions. The West Australian Indigenous Storybook project created storybooks in collaboration with Aboriginal Elders and a steering committee to share important cultural stories (25). This project began partly in response to negative media portrayals of Aboriginal Australians in media, recognizing the need to combat the detrimental health impacts of negative representation of Indigenous peoples in media (25, 26).

Accurate portrayal of contemporary AIAN peoples can do more than just engage an audience in health education and promotion, it can also serve to increase positive representations of Indigenous communities and peoples today, rather than the romanticized and harmful depictions of AIANs often seen in popular media (27). There is a woeful lack of accurate, respectful representation of AIAN peoples and communities in media, which contributes to severely limited views and understandings of AIAN identities and leads to harmful stereotypes (e.g., AI mascots) (27–29) and negative health, mental health, and academic outcomes for AIAN youth and adults (29–32). However, in both community and academic spaces, storytelling is a sacred responsibility that requires deep intentionality. As described by Mallory Whiteduck: “When we write, Native writers are responsible to our families, our communities, and the larger Native academic community. Our stories represent a fundamental love and respect for our homeland, and writing them ensures our children can return home regardless of their physical location. Through stories we can achieve decolonization by responding to past and ongoing oppression, while actively moving beyond it” (33). Storybooks hold promise as a multi-faceted resource to improve health and well-being through storytelling that expands modern representation of AIAN communities, peoples, and strengths. Further, the writing of storybooks requires dedication to community, and to the very act of storytelling. Stories cannot be separated from the storyteller, and therefore developing storybooks requires a deeply committed, intentional, and thoughtful writing team dedicated to not only responding to oppression but also to demonstrating active resistance and flourishing beyond oppression.

Considering that the inclusion of storytelling and other culturally appropriate materials and methods increase the effectiveness and sustainability of Indigenous public health projects (34), in March 2020, the Johns Hopkins Center for Indigenous Health (CIH) adapted *My Hero is You* (35). *My Hero is You* is a storybook aimed at helping children across the globe cope with the mental and social health impacts brought by the COVID-19 pandemic and developed by the Inter-Agency Standing Committee Reference Group on Mental Health and Psychosocial Support in Emergency Settings (36). The book’s approach of delivering guidance and teachings through storytelling made it a compelling candidate to address the pandemic’s impact on AIAN youth and communities (35). However, *My Hero is* You, lacked cultural specificity and important context unique to the experiences of AI/ANs during the COVID-19 pandemic. Therefore, the CIH convened a collaborative working group of AI/AN child health and development experts who represented diverse tribal affiliations, as well as professional and personal experience with AIAN children. The adaptation and dissemination of this book, *Our Smallest Warriors, Our Strongest Medicine: Overcoming COVID-19 (OSWOSM1),* is detailed in a publication by O’Keefe and colleagues (35).

After the release and distribution of *OSWOSM1*, the COVID-19 pandemic continued, and the public health response shifted from immediate crisis management to long-term, sustainable initiatives. To meet the ongoing and emergent needs of AIAN children and their caregivers in the changing pandemic landscape, the CIH created a culturally grounded sequel to *OSWOSM1*. This sequel, *Our Smallest Warriors, Our Strongest Medicine: Honoring Our Teachings during COVID-19 (OSWOSM2)*, was developed to provide continued support for mental and emotional health for AIAN children and their caregivers, promote updated public health guidance related to COVID-19, and to elevate and center Indigenous cultural strengths and teachings (37). To create the storybook, the CIH team (authors TM, FG, MC, AI, EH, and VO’K) re-convened an Indigenous collaborative workgroup (authors JA-B, JJ, LM, CA, and MR) from the first book, *OSWOSM1,* with the addition of an Indigenous child well-being researcher (author JS). The members of the collaborative workgroup (herein, workgroup) hold diverse Tribal affiliations and personal (i.e., many are parents themselves) and professional expertise in various aspects of child development and well-being. This manuscript describes the development and dissemination of *OSWOSM2*, a culturally grounded AIAN children’s storybook.

## Methods

Each member of our storybook development team brought their own unique cultural values, experiences, and expertise. Further each member had a unique experience with the COVID-19 pandemic and these experiences implicitly and explicitly shaped the creation of the storybook. In Table 1, authors who engaged in the development of the book reflect on their involvement with creating the storybook and the specific topics or experiences they hoped the book would represent. By communicating the hopes and intentions of our authorship team, we demonstrate that the authorship team engaged in storybook development with intentionality in the hopes that this book would not only respond to the oppression of the ongoing COVID-19 pandemic, but tell a story of survivance and thriving in the face of adversity.

**Table 1:** Reflections from members of the storybook development team.

Team member (Tribal affiliation if applicable)	Reflections
Tara Maudrie (Sault Ste. Marie Tribe of Chippewa Indians)	As an Anishinaabe woman, I have always found comfort and strength in my cultural teachings, especially during times of hardship. Through the storyline, I hoped to reinforce what our people have always known—that our strength lies in our connection to one another, land, and culture. As a child I had very little representation of Indigenous peoples in media and books and what representation of Indigenous peoples existed did not reflect my experience. My hope was that this book provided positive representation of Indigenous peoples and encouraged people to learn about and lean on their teachings during hard times.
Fiona Grubin	As a non-Indigenous person who strives to be an ally, I am grateful to support this important endeavor. I hoped to be a part of elevating and bringing the visions of the collaborative workgroup to life and, in doing so, contributing to more positive representation of Indigenous peoples in media. Along the way I appreciated the opportunity to learn from the group and the values and teachings that were discussed and included in the book.
Jessica Ullrich (Inupiaq, Nome Eskimo Community)	Children’s books are a wonderful way to learn and for parents to engage in conversations with their children about important life lessons and teachings. Stories are our way of teaching, healing, growing, and feeling. My hope with this book was for children to feel supported and connected through story, even as they were experiencing the challenges of navigating a pandemic.
Joshuaa Allison-Burbank (Diné and Acoma Pueblo)	Young Indigenous children interpret the world in unique ways. The way they see and understand the world reflects a worldview that prioritizes connection and kinship. During the pandemic, critical connections to family, community, and the land were disrupted and this altered the worldview of these young children. This book provided the opportunity for loved ones to openly discuss what was happening in their community and offered strategies on how to talk about traumatic experiences linked to the pandemic. Most importantly, the use of shared book reading is a powerful way for caregivers to bond with their children and offer comfort during through positive attachment. During a time of isolation and social distancing, offering children’s books and strategies for how to implement shared book reading was a special way to deliver much needed caregiver support.
Lisa Martin (Sault Ste. Marie Tribe of Chippewa Indians)	Teaching our young people how to deal with hard things, like grief, by honoring our teachings provides them with tools and options for coping. In this book I wanted to uplift approaches to dealing with hard things that were grounded in the beliefs and cultural practices of Indigenous communities.
Crystal Austin (Diné Nation)	In this book, I wanted to represent my experience as a parent and validate the experiences of other parents, it was difficult for many parents to navigate online and hybrid school. The safety checklist shared in the book is something I practice with my children, as it helped them to manage anxiety and to feel a sense of control, especially when so much of our lives felt out of control. I also wanted to highlight the good things even during hard times, like staying connected when we are physically apart. As Indigenous peoples, no one is ever truly alone. Our kinship within our communities gives us a sense of identity and reminds us that we are responsible for one another. This sense of connection enables our survival and our thriving as Indigenous peoples, even during hard times.
Joelle Joyner (Kauwets’a:ka)	I wanted to make sure there was representation of Indigenous peoples through diverse features, clothing, and environments in contemporary Indigenous communities through the illustrations I created for the book.
Marcy Ronyak (Confederated Tribes of the Colville Reservation)	Being a part of a unique opportunity to share public health messaging with Indigenous communities, ties closely to our storytelling practices. It was a highlight of my federal career, being able to work with an outstanding group of Indigenous allies who wanted to make a difference within a variety of cultural settings that supported a bond with caregivers, health education, and an opportunity to deliver messaging during a traumatic timeframe in our nation. The hope was that the book would provide an opportunity for connectedness during a time of isolation and social distancing.
Emily Haroz	It has been a true gift to work on these projects. I am not Indigenous, but I work every day to be an ally to Indigenous peoples and communities. Focusing on children provides me with hope for the future. Seeing contemporary Indigenous families and children in print is good for all kids to help them better understand the strength of Indigenous peoples and practices.
Victoria O’Keefe (Cherokee Nation/Seminole Nation)	As a Cherokee/Seminole woman, my community and cultural values shape how I live and everything I do, including collaborating on this storybook series. Through this book series, we have illuminated the inherent and continued Indigenous strengths, values, and traditions that wrap us in love, care, and protection, even during trying times like COVID-19. These strengths, values, and traditions have been passed down to us through our ancestors and will uplift our communities for generations to come.

### Storybook creation process

The development of the storyline for *OSWOSM2* took place over 6 weeks from January to February 2021 via iterative virtual meetings (i.e., we did not determine how many meetings would be held but met until we had achieved our goals). During the development process, the workgroup discussed rapid changes in the COVID-19 pandemic public health response since the development of *OSWOSM1* and how these changes have affected AIAN children and their caregivers. Through these discussions the workgroup considered numerous AIAN community strengths demonstrated during the COVID-19 pandemic, such as innovative public health responses (e.g., utilizing Tribal sovereignty to implement stay-at-home orders to reduce COVID-19 spread) and cultural values that promote community health and wellness (e.g., caring for Elders, safely maintaining social and cultural connection). The workgroup agreed it was important for the storybook characters to convey diverse experiences, like living in an urban or reservation environment, and to represent mixed racial and Tribal identities.

The team came to consensus to use a similar format to *OSWOSM1*. This included keeping the main characters, twins Tara and Virgil, using the four directions as a foundation for illustrating distinct pandemic experiences, including COVID-19-related public health guidance at the time of development, and harnessing cultural teachings throughout the book. Workgroup members recommended specific content to be represented in the story to mirror their own family and community experiences (e.g., drive-through birthday celebrations, receiving COVID-19 vaccines, and sending care packages to loved ones). In addition, the workgroup agreed to the importance of addressing children’s mental and emotional health in the storybook (e.g., by discussing coping strategies for grief and disconnection resulting from social distancing measures). Workgroup members provided specific examples of how cultural teachings offer frameworks and value systems that have supported the health of Indigenous communities since time immemorial. The workgroup agreed that honoring cultural teachings during the COVID-19 pandemic was an appropriate theme for the book as it reflected the way that many Indigenous communities across Turtle Island (i.e., North America) were responding to the pandemic, while grounding the story in traditional values and knowledge. For example, Indigenous communities in Wisconsin highlight four main themes that support health and wellness during (and before and after) the pandemic: “(1) helping my people, (2) honoring our elders, (3) self-determination, and (4) living in a good way” (38). Workgroup members discussed witnessing and learning about similar cultural teachings embedded in public health responses to the pandemic in their own and other tribal communities. The working group did not encounter any significant disagreements throughout the storybook development process. However, occasionally some suggestions for elements of the story (e.g., an animal) had different cultural meaning across working group members. When this occurred working group members discussed thoroughly the meaning of the suggested element within their respective cultures, before reaching a solution that had the intended meaning across the represented cultural groups within the working group.

Based on a storyboard collaboratively developed from meetings with the workgroup, the CIH team (authors TM, VO’K, and FG) drafted the initial storyline text. We incorporated feedback from caregivers who read *OSWOSM1* with their children. Caregivers recommended less text per page to help children better engage with the book and providing guidance to consider reading the book in multiple sessions. Facilitated by the CIH team, versions of the storyline were reviewed iteratively with the workgroup until consensus was reached on the book’s final text. We did this through regular virtual meetings where the draft storybook content was presented, reviewed, and discussed by workgroup members until everyone agreed that the draft was final. Next, AI artist and workgroup member (author JJ) created illustrations to accompany the final text over 3 months (March–May 2021). Illustrations were reviewed by the workgroup to ensure resonated with the diversity of AIAN experiences represented in the storyline. Following this process, the CIH team worked to compile the final text and illustrations together to create *OSWOSM2*.

### Dissemination plans

The CIH team planned to facilitate all dissemination of *OSWOSM2* using similar strategies employed for distributing *OSWOSM1*, including online through the CIH webpage, CIH social media platforms (Facebook, Instagram, and Twitter), and emails to all Tribal, Inter-Tribal, Urban Indian Health Programs, school-based, clinic-based, and home-visiting organizations who received print copies of *OSWOSM1* (35). In keeping with dissemination efforts of *OSWOSM1*, we also made an online request form available for Native-serving organizations to request bulk orders of print copies of the book for wider distribution within their community. These requests were shipped out on a first come, first serve basis as funding allowed. Distributing print copies of the book was important to the CIH team, as at least 31% of people living on Tribal reservations report having no or unreliable internet connection access (39). Bulk shipments were sent to CIH offices in the Southwest and Midwest where the books were shared with communities through programs and community networks. The final *OSWOSM2* book and accompanying resources were made available for free download on the CIH website.^1^ We implemented a series of social media posts to advertise availability of the book and resources and to celebrate the content of the book.

### Caregiver survey

In conjunction with *OSWOSM2* dissemination, we promoted an optional survey intended for parents and caregivers to complete after reading the book with their children. The goal of this survey was to briefly assess the general impact and reach the book had on AIAN children and families. The caregiver survey was hosted using Qualtrics (40), an online data collection platform, and distributed via links, and QR codes embedded in emails and social media posts about the storybook, as well as on the back of all print copies of the storybooks. 13 survey questions assessed overall satisfaction with the book, whether new information and skills related to the COVID-19 pandemic or mental health coping were learned through reading the book, satisfaction with the book’s cultural teachings and illustration of modern day AIAN people, and whether respondents would be interested in future books of this nature. Two open-ended questions asked about (1) what topics respondents might like to see in future books and (2) whether respondents had any additional feedback to share about the book. Respondents who completed the survey were entered for a chance to win one of five $20 visa gift cards. The Johns Hopkins Bloomberg School of Public Health Institutional Review Board determined this survey as not human subjects research and did not require IRB oversight (BSPH IRB #00016998). We did not seek specific Tribal approvals for this survey as it was intended, like the book, to reach a broad audience of any AIAN people. Additionally, participation in the survey was voluntary and the broad goal was to generally learn about how the book was received to help us improve future, similar programming and resources.

## Results

### Our Smallest Warriors, Our Strongest Medicine: Honoring Our Teachings during COVID-19

**Figure 1:**
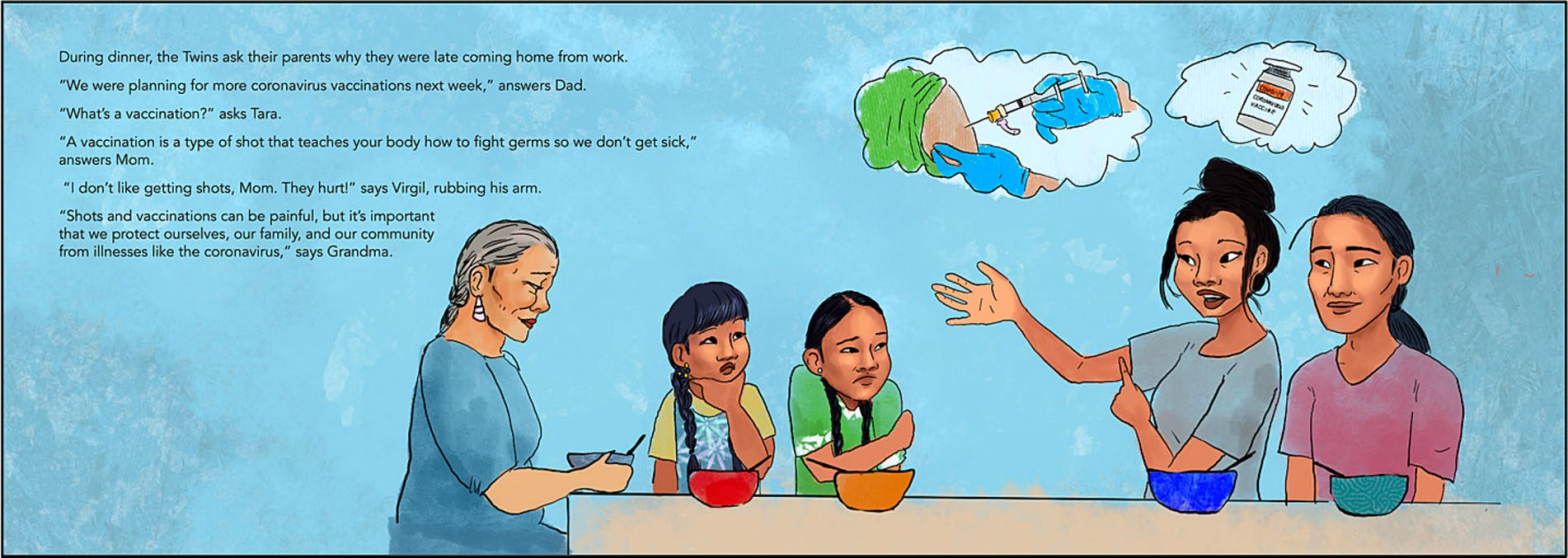
OSWOSM2 storybook page about vaccines. Image credit: JJ.

**Figure 2:**
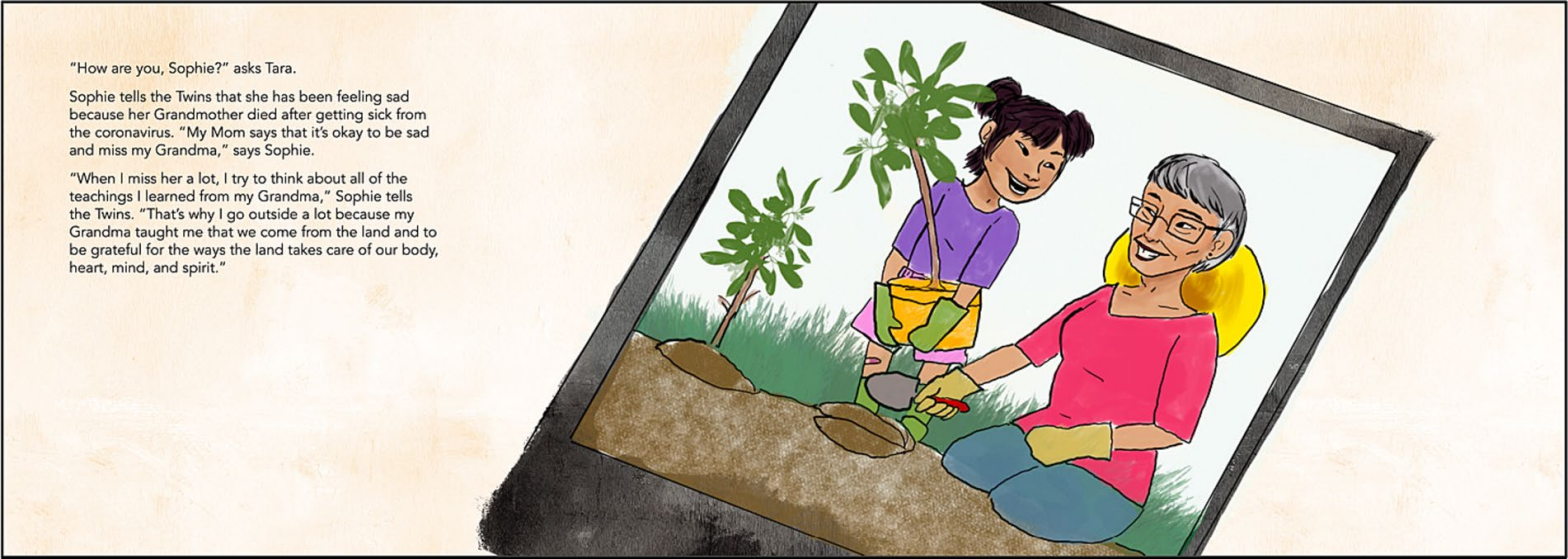
OSWOSM2 storybook page about grief, healing, and land. Image credit: JJ.

The final *OSWOSM2* storybook contains 57 pages, with 29 pages that include storyline text and 46 pages with full-page illustrations. Figures 1, 2 below illustrate examples COVID-19 messaging and illustrations from the final storybook. An online toolkit accompanied the book including: six coloring pages, six traditional language activities, a two page resource for reading and talking about the pandemic with children, a vocabulary page with relevant words from the story, and a worksheet to encourage reflection on the readers’ cultural teachings. The storyline presented in *OSWOSM2* provides public health information and mental health education for children and parents that is grounded in the strengths and teachings of Indigenous communities.

The reader accompanies twins Tara and Virgil as they encounter emerging public health initiatives to mitigate the effects of the COVID-19 pandemic while maintaining social connections with friends and family (characters were introduced in *OSWOSM1*). Through their family the twins learn about vaccination efforts in their own community, and they learn from their friends located in the four directions about how they are staying connected and coping with the pandemic. Each of the four friends offers a different teaching accompanied by practical advice on how children can apply the teaching to promote well-being. For example, the Twins’ friend Daniel shares through a letter how COVID-19 continues to impact his family’s life and that when he feels scared or worried, he can talk about his feelings with family members. He says that his mom taught him that he carries the strength of his ancestors and that helps him feel strong. Later, the twins share with their family that Daniel taught them it is okay to feel all of their emotions, which reflects cultural values of holistically honoring all aspects of health, including one’s emotions. The story concludes with the twins sharing with their family what they learned from each of their friends’ experiences, cultural teachings, and their own message of hope for the future.

### Dissemination

**Figure 3:**
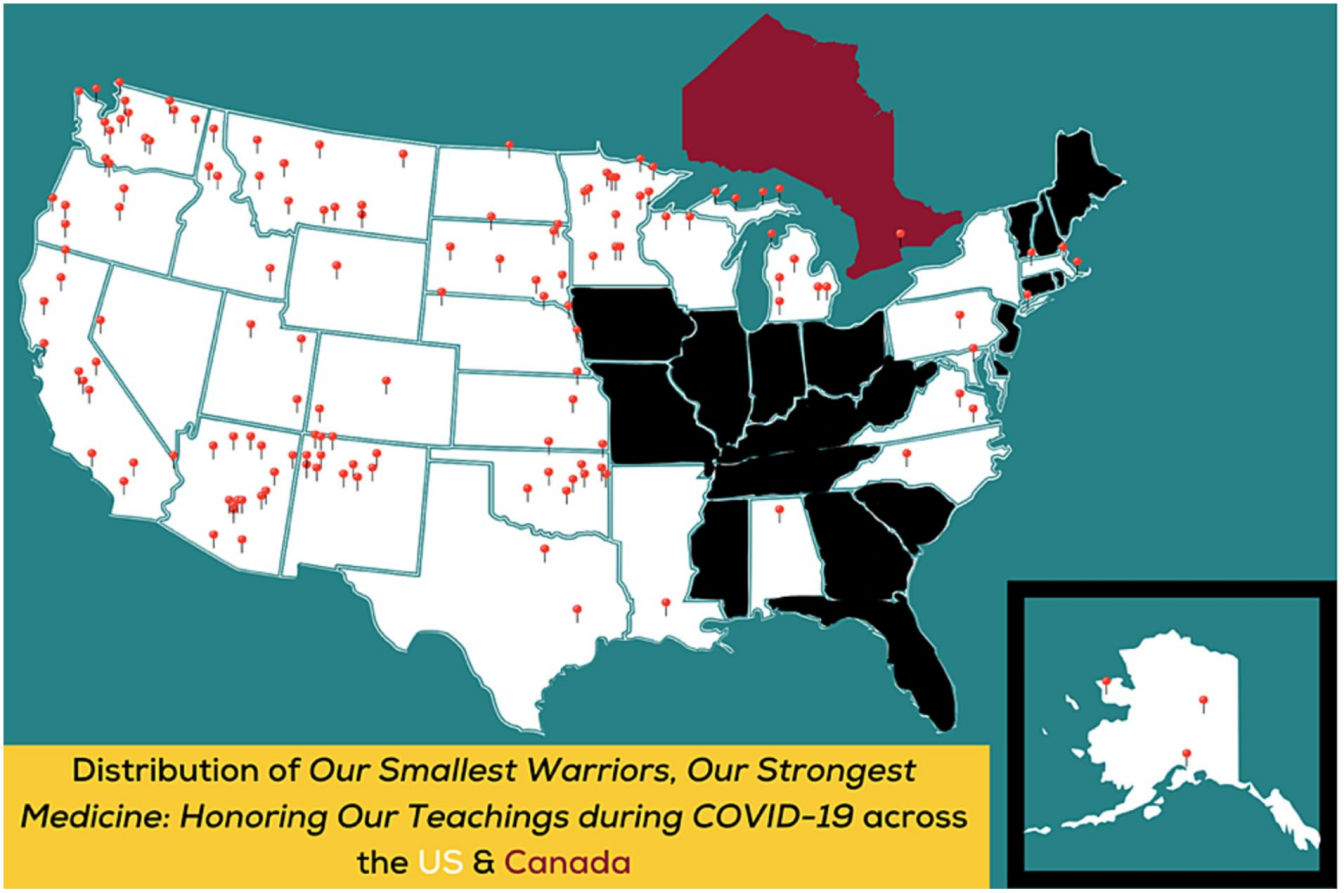
Distribution of OSWOSM2.

*Our Smallest Warriors, Our Strongest Medicine: Honoring Our Teachings during COVID-19* books reached a wide variety of communities. In total, 50,024 print copies of books were distributed across all 12 of the Indian Health Service (IHS) regional areas in the United States, reaching 28 states, 105 tribes, and two First Nations communities in Canada (Figure 3). The books were shared with Indigenous families by 60 different urban and Inter-Tribal organizations, 35 IHS clinics, 40 educational organizations, 20 Head Start programs (most of them Tribal Head Start programs), and 12 Family Spirit® Tribal home visiting affiliates (41). Between June 2021 and February 2023, CIH developed and published 14 social media posts promoting *OSWOSM2* on CIH accounts. These posts achieved a combined total reach of 12,284 impressions on Facebook, 12,274 impressions on Twitter, and 3,798 impressions on Instagram. The CIH was undergoing a re-branding and re-naming (formerly Center for American Indian Health) transition during the time books were being distributed which unfortunately meant we were unable to access relevant analytic information from the CIH website related to electronic downloads of the book.

### Caregiver survey results

A total of 34 responses to the caregiver survey were determined to be valid for analysis. We implemented survey protections such as reCAPTCHA and responses with a high score for risk of being fraudulent were not analyzed. Of these 34 included responses, most respondents (*n* = 30) reported that a parent, caregiver, or other adult read the book with a child, and five of these people also reported the child had read the book on their own. When asked to rate their satisfaction with the book from 0 (not at all) to 5 (completely), the average response from *n* = 23 respondents was 4.8, indicating very high overall satisfaction. No responses below a four were received for this question. Satisfaction with the book’s cultural teachings and representation of AIAN peoples through text and illustration was also high. 94% of respondents reported being extremely or somewhat satisfied with the cultural teachings presented in the books, 90% reported extremely or somewhat satisfied with the book’s illustration and text depicting and describing modern AIAN peoples.

The data also show that most families who completed the survey learned new information about mental health coping and preventing COVID-19 from reading the book. 90% of respondents said that they had probably or definitely learned something about mental health coping, while 79% reported probably or definitely learning something about COVID-19 prevention. All but one respondent shared they would be interested in future books on other health/wellness topics, and 16 people shared open-ended topic ideas for future books including mental health, language books, diabetes education, and wellness. Lastly, *n* = 12 respondents noted open-ended feedback about the book, all of which were positive and featured messages such as how much children enjoyed the books, appreciation for the Tribal traditions reflected in the book, and gratitude for the book. One respondent shared, “My kids love the illustrations of the book and said they were excited to see Natives like themselves. They mentioned that was them on the book or it was their cousins. They connected with the book and told their friends about it.” Another caregiver shared, “Thank you. The book made a lot of children very happy when they received it. It showed that we all can get COVID-19, and that it reflected tribal traditions and community.”

## Discussion

The *OSWOSM* storybooks represent a resurgence and revitalization of Indigenous ways of knowing and being, which cultivate strength and innovation (42). Through developing *OSWOSM2* in collaboration with an Indigenous Workgroup, we used storytelling as a creative way to share public health information with children and their caregivers. As researchers who employ a Community-Based Participatory Research (CBPR) approach, developing and distributing the book to AIAN children and families was a form of creative dissemination and public service. Responding to the health needs of our communities is emblematic of our duty care for our communities in line with a CBPR approach. Our evaluation of the book showed that readers reported high satisfaction with the book and indicated that they learned new things about mental health coping and preventing COVID-19, further highlighting the power of creative dissemination and positive representation of Indigenous peoples in media.

Since time immemorial, Indigenous communities have told stories as a way to pass knowledge and teachings intergenerationally, to facilitate connectedness, and to share experiences across time (43). Engaging with storytelling is an important part of AIAN children’s development as it integrates dynamic learning with socialization and passes on cultural values and teachings in an engaging and developmentally appropriate way (44). Indigenous storytellers often tell the same stories multiple times, as with each telling there is something new to learn based on the listener’s growth, development, understanding, and life experience (45). Storytelling is both a practice and an Indigenous research method that provides an opportunity for children and their caregivers to actively engage in learning as they gather information from a story and return to that information iteratively as they navigate similar situations in their day to day life, encouraging them to continue to connect more deeply to different aspects of the story throughout time (43). Within a story are embedded values, emotions, behavioral actions, relational connections, and teachings that guide children on ways to live and be in the world in response to life’s joys, celebrations, and challenges.

Using a storytelling approach in *OSWOSM2* was not only responsive to the needs of children and their caregivers for easily understandable public health guidance, but reflective of the power of AIAN storytelling and Indigenous research methods to provide validation, and to facilitate connection, reflection, and discussion (43). An Ojibwe storyteller, writer, and scholar, Leanne Betasamosake Simpson, elaborates on the power of Indigenous storytelling: “For me, storytelling is a way of connecting to the land, and it is a way of connecting with the past, and it is also a way of connecting to the future…People are asked to see themselves in the story, to carry the story, to work with the story, and to find their own meaning within a story. Telling my children stories is like planting little seeds inside them. What I hope as a parent is that I have given my kids this garden of stories and then when life brings them challenges, they will have this body of knowledge that will provide them with comfort” (45). The format, content, and illustrations of *OSWOSM2* encourage children and their caregivers to see themselves in the story—these efforts were validated by caregiver responses to surveys which highlighted that children saw aspects of themselves and their experiences mirrored in the story. Integrating aspects of Indigenous storytelling with public health guidance encouraged children to process their emotions surrounding the pandemic and emphasized the power of cultural teachings to provide comfort and guidance as children navigate challenges. *OSWOSM2* builds on the rich tradition of AIAN storytelling through a storybook to provide children with a space to think, feel, learn, and gain wisdom, both from ancestral teachings and through their own family and community’s response to the pandemic.

Efforts to mitigate the spread of COVID-19 often required communities to implement closures of schools and community programs. As public health understanding of the pandemic advanced, it became evident that a full return to in-person school would be delayed and that support was needed to help children navigate difficult learning and life routines (46). *OSWOSM2* responds to the needs of AIAN children and their caregivers by embedding child mental health practices in the story like spending time outside and practicing sharing feelings. The efforts to develop a culturally grounded storybook were aligned with our teams’ values to honor community strengths and serve community needs and are one example of putting CBPR principles into practice (47, 48). The storybook is a creative, fast way to distribute much-needed mental health support to AIAN children that elevates protective AIAN values and beliefs. The storybook both represents and fosters creative expression, which contributes to positive connection with individual and community identities, provides a platform to share information about coping skills, and can augment the CBPR process (49). *OSWOSM2* responded to community feedback from *OSWOSM1*, integrating specific recommendations from our informal evaluation to meet the needs of AIAN children and their caregivers. Rapid dissemination of *OSWOSM* books during the COVID-19 pandemic aligns with CBPR values of being action-oriented with specific intent to benefit the community (47).

This project represents an example of an innovative approach to public health communication that is aligned with Indigenous methodologies and strengths. The second book in the *Our Smallest Warriors* series builds on the strengths of the previous book and met the demand for COVID-19 resources specifically for AIAN children that are reflective of evolving public health guidance. However, *OSWOSM2* is a culturally built and culturally grounded resource, where *OSWOSM1* is a cultural adaptation of an existing storybook. Culturally grounded resources offer significant advantages for Indigenous communities as they are created from community values and strengths, and therefore have stronger acceptability to community (50, 51). Despite its strengths, there are important limitations to consider when interpreting the results. While the creation of the book was done in a way to be inclusive and representative of many Indigenous Nations, there was no feasible way to incorporate all 574 federally recognized Tribal Nations, nor all state recognized tribes in a comprehensive way. Images and storylines may not generalize to all communities and peoples. The caregiver survey was voluntary and based on convenience sampling. This approach introduces potential sampling and selection bias, and we cannot be sure our results generalize to all people who interacted with the book and associated materials. However, as this was an example of public health practice, generalizability of results was not the intention. Rather, our goal with the survey and results is to be transparent and accountable to the greater community and to continuously improve our processes for future work related to storytelling as a public health modality. Further, our evaluation efforts did not specifically assess the acceptability or impact of messaging on children or their caregivers. Future evaluation efforts of children’s storybooks may consider more robust evaluation efforts in order to make stronger conclusions about the impacts of storybook messaging.

In Indigenous communities storytelling is an intergenerational practice that builds on cultural traditions and teachings and encourages active learning. Our use of a storybook series brings this powerful tradition to the daily lives of AIAN children and families while serving as an Indigenous public health intervention. This storybook not only provided a health resource during a specific point in time (i.e., the COVID-19 pandemic), but created an opportunity for children, families, and communities to uplift and carry on cultural values and traditions now and in the future. We hope the *Our Smallest Warriors* series will inspire future culturally grounded efforts that braid Indigenous methodologies with public health dissemination.

## Data availability statement

The datasets presented in this article are not readily available because data are not publicly available. Requests to access the datasets should be directed to TM, tmaudri1@jhu.edu.

## Ethics statement

Ethical approval was not required for the study involving humans in accordance with the local legislation and institutional requirements. The studies were conducted in accordance with the local legislation and institutional requirements. Written informed consent to participate in this study was not required from the participants in accordance with the national legislation and the institutional requirements.

## Author contributions

TM: Conceptualization, Data curation, Formal analysis, Investigation, Methodology, Project administration, Resources, Writing – original draft, Writing – review & editing, Validation. FG: Conceptualization, Data curation, Formal analysis, Funding acquisition, Project administration, Resources, Writing – original draft, Writing – review & editing. MC: Data curation, Formal analysis, Project administration, Validation, Writing – original draft. JV: Data curation, Formal analysis, Writing – original draft. JS: Conceptualization, Writing – original draft. JA-B: Conceptualization, Writing – original draft. LM: Conceptualization, Writing – review & editing. CA: Conceptualization, Writing – review & editing. JJ: Conceptualization, Visualization, Writing – review & editing. MR: Conceptualization, Funding acquisition, Writing – review & editing. KM: Writing – review & editing, Visualization. AI: Writing – review & editing, Project administration. EH: Conceptualization, Funding acquisition, Supervision, Writing – original draft, Writing – review & editing. VO’K: Conceptualization, Data curation, Funding acquisition, Investigation, Supervision, Writing – original draft, Writing – review & editing.

## Footnotes

^1^ bit.ly/cih-oswosm2
